# The single pregnancy predicting model of 1 minute Apgar score less than 7 after preterm birth: A retrospective study

**DOI:** 10.1371/journal.pone.0279385

**Published:** 2022-12-22

**Authors:** Xi-Shi Lin, Xin-Yun Peng, Meng-Meng Yang, Lin-li Ning, Yu-Wei Shao, Ying Jiang, Su-Wen Feng, Qiong Luo

**Affiliations:** 1 Department of Obstetrics, Women’s Hospital, Zhejiang University, School of Medicine, Hangzhou, China; 2 Hangzhou Fuyang Women and Children Hospital, Hangzhou, China; 3 Changxing Women and Children’s Hospital, Huzhou, China; Universidade de Sao Paulo Instituto de Ciencias Biomedicas, BRAZIL

## Abstract

Preterm delivery is greatly associated with perinatal mortality and morbidity, while there is no objective way to identify high-risk newborns currently. This study aimed at discovering the risk factor for Apgar score less than 7 at 1 minute of preterm neonates born with vaginal delivery. A retrospective study was performed in single pregnancy women with a vaginal delivery before 37 weeks of gestation. All the preterm infants were categorized into three types: very preterm birth (28 to 32 weeks), moderate preterm birth (32 to 34 weeks) and late preterm birth (34 to 37 weeks). Risk factors were identified through logistic regression analysis in every category of newborns mentioned above. And the receiver operating characteristic analysis was used in continuous variables to determine the best threshold of the outcome. On the basis of the selected factors, the predicting models are created and its prognosticating ability is compared by the area under the curve. A nomogram was established for the proved best model. A total of 981 cases were investigated, of whom 55 were found with 1 min Apgar scores less than 7. The nomogram was set for the predicting model with larger area under the receiver operating characteristic curve, of which is 0.742(95% confidence interval = 0.670–0.805) in very preterm birth, with the variables of first and second labor stage(> = 1.6 hours), birthweight and MgSO4(magnesium sulfate), and is 0.807(95% confidence interval = 0.776–0.837) in late preterm birth, with the variables of second labor stage(> = 1.23 hours), birthweight, a history of previous cesarean delivery, fetal distress and placental abruption. The combination of first and second labor stage, newborn weight and MgSO4 use can predict 74.2% of 1 minute Apgar score < 7 in very preterm neonates. And 80.7% high-risk infants can be identified when second labor stage, newborn weight, VBAC (vaginal birth after cesarean) and the occur of placental abruption and fetal distress were combined in the predicting model for late preterm birth. These predicting models would bring out great assistance towards obstetricians and reduce unnecessary adverse fetal outcomes.

## Introduction

The prevalence of preterm delivery appears to be showing an increasing trend globally [1–3]. It could be due to advanced maternal age and to widespread use of assisted reproductive technology [4–7]. Besides, it had been demonstrated that preterm delivery greatly contribute to perinatal mortality and morbidity [8, 9]. More than 1 million infants had been witnessed to die before the age of 5 every year because of prematurity birth [10]. Premature infants also encountered higher risk of short-term complications, like respiratory syndromes and intraventricular hemorrhage, and long-term morbidity, including neurologic disabilities and delayed school performances [11, 12].

Gestational age at delivery is crucial to neonatal survival and morbidity [13]. So preterm birth generally be classified into three categories: very preterm birth (28 to 32 weeks), moderate preterm birth (32 to 34 weeks) and late preterm birth (34 to 37 weeks) [14]. Although moderate to late preterm birth take over a majority part of all preterm birth [9, 15], very preterm delivery comprised over half of infant death [16]. It is of great importance to enhance the identification of high-risk infants among preterm deliveries, so that timely intervention can be put into action to improve perinatal outcomes.

The Apgar score is generally used as a quantitative standard for assessing newborns status nowadays [17]. It presents the neonates status at birth and the probability of death [18]. The score value underneath 7 had been proved to be associated with various short-term complications including hypoxic ischemic encephalopathy, hypoglycemia and aspiration complications [19]. It also connected with long-term complications like sensorineural hearing loss and pediatric ophthalmic morbidity [19–21]. Hence it is widely-used to detect high-risk newborns [22, 23].

Previous studies had found several risk factors about Apgar score less than 7, involving the duration of labor stage [24], maternal BMI(body mass index) and the history of caesarean section [25–27]. Gestational age had also been noted as an influencing factor [23, 28]. Additionally, preterm birth had been documented to be a prominent relevant factor for Apgar score <7 [28]. However, to the best of our knowledge, study on discriminating the risk of low Apgar score targeting solely at preterm delivery is scarce.

Consequently, our study aimed at identifying risk factors related to a low Apgar score specializing in premature deliveries, and further establishing a predicting model.

## Methods

### Study design and population

To identify the risk factors for 1 minute Apgar score less than 7 amongst preterm neonates delivered through the vaginal route. A retrospective study using clinical data was carried out in three Chinese maternity units. Between 1 Jun 2016 and 30 Jun 2019, women who had preterm birth at three hospital centers (Women’s Hospital, Zhejiang University School of Medicine, Changxing Maternal and Child Health Hospital and Fuyang Maternal and Child Health Hospital) were enrolled in this retrospective study. This study was approved by the Clinical Research Ethics Committee of Women’s Hospital Zhejiang University School of Medicine. The inclusion criteria of our research were: (1) singleton pregnancy; (2) the accessibility of integrated medical records; (3) giving birth at cephalic position; (4) delivery time between 28–37 week. And the exclusion criteria were as follows: (1) abnormal fetal positions, such as breech or shoulder presentation; (2) patients complicated by the following complications: pre-gestational diabetes, hyperthyroidism; (4) uterine malformation like unicornuate uterus, uterus duplex; (5) cervical surgery history: cervical cerclage, cervical conization.

### Data resource

All the demographic and obstetric data were obtained from medical records in three centers. The collected demographic data included gestational week at delivery, maternal age, preconception and prenatal body mass index (BMI), obstetrics history including cesarean delivery. We also retrieved all the information about maternal complications: hypertension disorders (HDP), gestational diabetes mellitus (GDM), intrahepatic cholestasis of pregnancy (ICP), abnormal amniotic fluid, placenta abruption, preterm premature rupture of membranes (PPROM), intrauterine infection, fetal distress (judged by the abnormalities in cardiotocography), postpartum hemorrhage (PPH). Besides, we summarized medical administration for every patient, which contained the use of magnesium sulfate (MgSO4), dexamethasone before delivery. Finally, the collected neonatal data were as follows: Apgar score after 1 and 5 minutes, delivery records, birthweight and the admission to the neonatal intensive care unit (NICU).

### Statistical analysis

Statistical analyses were performed using R software (Version 3.6.0; https://www.R-project.org) and IBM SPSS statistics software version 26(IBM, Armonk, NY). We used mean and standard deviation (SD) to represent numerical variables, and percentages to represent qualitative variables. Using an analysis of variance (ANOVA) or non-parametric test (such as Mann-Whitney U test) to compare differences in numerical variables. Comparison of qualitative variables between case-control groups was performed using a Chi-square test. The best threshold of qualitative variables was predicted from the best sensitivity and specificity by the receiver operating characteristic (ROC) analysis. The DeLong’s test was used to examine the statistical differences of ROC curves. The prediction models were established based on the results of logistic regression analyses. The nomogram of predicting models was formulated by R software. Discrimination of the prediction models were assessed by the area under the ROC curve (AUC). Calibration of the predicting models were adopted by calibration curves.

### Ethics approval and consent to participate

Our study was designed in accordance with the Declaration of Helsinki. The study protocol was approved by the Clinical Research Ethics Committee of Women’s Hospital Zhejiang University School of Medicine (IRB-20210239-R). And the exemption from informed consent was approved by the committee for that the nature of the study was extracting and analyzing the existing clinical data, which had minimal influence in the study population. Also, we maintained confidentiality through excluding names or any other personal identifiers during data collection.

## Results

### Patients’ characteristics and univariable analysis

There were 981 cases enrolled in our study. Among them, 55 were found with 1 min Apgar scores less than 7, while 14 with 5 min Apgar scores less than 7. According to this, the population were divided into LOW-APGAR group and NORMAL-APGAR group. From 18 study variables presented in Table 1, 12 variables selected in the LOW-APGAR group exhibited statistically significant difference compared with the normal one (P<0.05), which included pre-conception BMI, vaginal birth after cesarean (VBAC), PPROM, placental abruption, intrauterine infection, fetal distress, MgSO4 use, dexamethasone use, first labor stage, second labor stage, first and second stage of labor (the sum of the first and second labor stage), newborn weight. There were no significant differences in the following variables: primiparas, maternal age, prenatal BMI and gestational complications like GDM, HDP, ICP. But the occurrence of PPH seemed to be increased among those women in LOW-APGAR group.

**Table 1:** Demographic and obstetrical parameters.

1 min Apgar scores of no more than 7				5 min Apgar scores of no more than 7			
	Control	Cases	P-value		Control	Cases	P-value
N	926	55			966	14	
Age			0.811				0.923
<35	750 (81.0%)	44 (80.0%)			781 (80.8%)	12 (85.7%)	
35–40	152 (16.4%)	9 (16.4%)			159 (16.5%)	2 (14.3%)	
>40	24 (2.6%)	2 (3.6%)			26 (2.7%)	0 (0.0%)	
BMI before pregnancy	812	49	0.005		850	11	0.299
<18.5	166 (20.4%)	13(26.5%)			176 (20.7%)	3 (27.3%)	
18.5< = BMI<24	520 (64.0%)	22 (44.9%)			537 (63.2%)	5 (45.5%)	
24< = BMI<28	101 (12.4%)	8 (16.3%)			107 (12.6%)	2 (18.2%)	
> = 28	25 (3.1%)	6 (12.2%)			30 (3.5%)	1 (9.1%)	
BMI before delivery	702	42	0.240		733	10	0.793
<18.5	5 (0.7%)	0 (0.0%)			5 (0.7%)	0 (0.0%)	
18.5< = BMI<24	202 (28.8%)	14 (33.3%)			212 (28.9%)	4 (40.0%)	
24< = BMI<28	317 (45.2%)	13 (31.0%)			326 (44.5%)	4 (40.0%)	
> = 28	178 (25.4%)	15 (35.7%)			190 (25.9%)	2 (20.0%)	
Primipara			0.653				0.681
No	285 (30.8%)	15 (27.3%)			300 (31.1%)	0 (0.0%)	
Yes	641 (69.2%)	40 (72.7%)			666 (68.9%)	14 (100.0%)	
GDM			0.481				0.491
No	750 (81.0%)	42 (76.4%)			778 (80.5%)	13 (92.9%)	
Yes	176 (19.0%)	13 (23.6%)			188 (19.5%)	1 (7.1%)	
HDP			0.874				0.681
No	893 (96.4%)	52 (94.5%)			930 (96.3%)	14 (100.0%)	
Yes	33 (3.6%)	3 (5.5%)			36 (3.7%)	0 (0.0%)	
ICP	926	55	0.632				1.000
No	913 (98.6%)	55 (100.0%)			656 (98.4%)	7 (100.0%)	
Yes	13 (1.4%)	0 (0.0%)			11 (1.6%)	0 (0.0%)	
VBAC			<0.001				<0.001
No	872 (94.2%)	43 (78.2%)			906 (93.8%)	8 (57.1%)	
Yes	54 (5.8%)	12 (21.8%)			60 (6.2%)	6 (42.9%)	
PPROM	926	55	0.001				0.162
No	332 (35.9%)	32 (58.2%)			356 (36.9%)	8 (57.1%)	
Yes	594 (64.1%)	23 (41.8%)			610 (63.1%)	6 (42.9%)	
Placental abruption	926	55	<0.001				0.648
No	841 (90.8%)	39 (70.9%)			867 (89.8%)	12 (85.7%)	
Yes	85 (9.2%)	16 (29.1%)			99 (10.2%)	2 (14.3%)	
Intrauterine Infection	925	51	0.009		963	12	0.588
No	865(93.5%)	42(82.4%)			895 (92.9%)	11 (91.7%)	
Yes	60 (6.5%)	9 (17.6%)			68 (7.1%)	1 (8.3%)	
Fetal Distress	924	55	0.006		964	14	0.032
No	881 (95.3%)	47 (85.5%)			916 (95.0%)	11 (78.6%)	
Yes	43 (4.7%)	8 (14.5%)			48 (5.0%)	3 (21.4%)	
MgSO4	925	55	<0.001		965	14	<0.001
No	601 (65.0%)	19(34.5%)			619 (64.1%)	1 (7.1%)	
Yes	324 (35.0%)	36 (65.5%)			346 (35.9%)	13 (92.9%)	
Dexamethasone	926	54	<0.001		965	14	<0.001
No	551 (59.5%)	17 (31.5%)			567 (58.8%)	1 (7.1%)	
Yes	375 (40.5%)	37 (68.5%)			398 (41.2%)	13 (92.9%)	
PPH	925	51	0.042		963	12	0.038
No	903 (97.6%)	41 (92.2%)			939 (97.5%)	10 (83.3%)	
Yes	22 (2.4%)	4 (7.8%)			24 (2.5%)	2 (16.7%)	
NICU			<0.001				0.002
No	342 (36.9%)	1 (1.8%)			343 (35.5%)	0 (0.0%)	
Yes	583(63.0%)	54(98.2%)			623 (64.5%)	14 (100.0%)	
First stage of labor (hours)							
28-31w+6 (148/27)	4.56 ± 4.07	4.79 ± 3.32	0.784	28-33w+6 (295/11)	4.74 ± 3.95	4.81 ± 3.87	0.954
32-33w+6 (123/8)	5.03 ± 3.99	3.72 ± 2.48	0.363				
34-36w+6 (655/20)	5.78 ± 4.38	7.86 ± 7.48	0.042	34-36w+6 (671/3)	5.79 ± 4.43	15.41 ± 11.07	<0.001
Second stage of labor (hours)							
28-31w+6 (148/27)	0.22 ± 0.30	0.37 ± 0.52	0.044	28-33w+6 (295/11)	0.27 ± 0.33	0.24 ± 0.23	0.764
32-33w+6 (123/8)	0.31 ± 0.31	0.16 ± 0.09	0.192				
34-36w+6 (655/20)	0.62 ± 0.63	0.83 ± 0.75	0.158	34-36w+6 (671/3)	0.63 ± 0.64	0.85 ± 1.03	0.547
First and second stage of labor (hours)							
28-31w+6 (148/27)	4.78 ± 4.07	7.61 ± 12.44	0.028	28-33w+6 (295/11)	5.01 ± 3.98	11.07 ± 19.16	<0.001
32-33w+6 (123/8)	5.33 ± 4.07	3.88 ± 2.44	0.321				
34-36w+6 (655/20)	6.40 ± 4.56	8.69 ± 7.66	0.032	34-36w+6 (671/3)	6.41 ± 4.60	16.26 ± 11.73	<0.001
Newborn weight (g)							
28-31w+6 (148/27)	1495.51 ±273.67	41319.26 ±219.75	0.002	28-33w+6 (295/11)	1724.42 ± 398.85	1354.09 ± 241.81	0.002
32-33w+6 (123/8)	2031.02 ±303.81	2103.75 ±250.03	0.509				
34-36w+6(655/20)	2647.07 ± 357.49	2607.00 ±550.28	0.628	34-36w+6 (671/3)	2647.48 ± 364.59	2396.67 ± 87.37	0.234

### ROC analysis for continuous predictors

We performed the ROC analysis for the 4 selected continuous variables to determine the optimal threshold. The results for 1 min Apgar scores of no more than 7 were presented in Table 2. We did stratified analysis by gestational week. The optimal cut-off of first labor stage in the divided groups were 2.67, 5.33 and 7.4 hours, respectively. For newborn weight, the best threshold was 1370, 2120, 2350 grams, respectively. As for low 5-min Apgar score group, no meaningful threshold can be found, which may be due to lacking positive cases. So further analysis was only performed for the low 1-min Apgar score group.

**Table 2:** ROC analysis for continuous predictors of 1 min Apgar scores of no more than 7.

		Best threshold	AUC	95%CI lower	95%CI upper	Specificity	Sensitivity
1 min Apgar scores of no more than 7 (28-31w+6)	First stage of labor (hours)	2.67	0.545	0.468	0.621	0.395	0.778
	First and second stage of labor (hours)	1.6	0.593	0.516	0.667	0.238	0.963
	Newborn weight (g)	1370	0.678	0.603	0.747	0.630	0.653
	Second stage of labor (hours)	0.13	0.576	0.499	0.650	0.599	0.593
1 min Apgar scores of no more than 7 (32-33w+6)	First stage of labor (hours)	5.33	0.579	0.490	0.665	0.358	0.875
	First and second stage of labor (hours)	5.47	0.587	0.498	0.673	0.382	0.875
	Newborn weight (g)	2120	0.584	0.495	0.670	0.667	0.625
	Second stage of labor (hours)	0.23	0.613	0.524	0.697	0.415	0.875
1 min Apgar scores of no more than 7 (34-36w+6)	First stage of labor (hours)	7.4	0.552	0.513	0.590	0.733	0.450
	First and second stage of labor (hours)	8.95	0.567	0.529	0.605	0.785	0.400
	Newborn weight (g)	2350	0.564	0.526	0.602	0.809	0.500
	Second stage of labor (hours)	1.23	0.555	0.517	0.593	0.864	0.400

### Multivariate analysis

In the multivariate analyses for 1 min Apgar score of no more than 7 in 28- to 32-week-old infants (Table 3), we found that when first labor stage prolonged more than 1.6 hours, the risk of low Apgar score increased by 7.298 times (95% CI = 0.953–55.872; P = 0.011). Compared with those who administrated MgSO4 before delivery, the risk of low Apgar score enhanced by 6.390 times in infants without this history (95% CI = 0.832–49.053; P = 0.020).

**Table 3:** The multivariate analysis for 1-min Apgar score<7 in very and late preterm birth infants.

Risk factor	Odds Ratio (95%CI)	*P*-value
28- to 31^+6^-week-old infants		
First and second stage of labor (hours)		
<1.6	Reference	
> = 1.6	7.298 (0.953–55.872)	0.011
Newborn weight (grams)		
< = 1370	Reference	
>1370	0.309(0.132–0.725)	0.007
MgSO4		
Yes	Reference	
No	6.390(0.832–49.053)	0.020
34- to 36^+6^-week-old infants		
Second stage of labor (hours)		
<1.23	Reference	
> = 1.23	4.132 (1.644–10.385)	0.003
Newborn weight (grams)		
>2350	Reference	
< = 2350	4.240(1.727–10.407)	0.002
VBAC		
No	Reference	
Yes	4.849(1.327–17.721)	0.017
Fetal Distress		
No	Reference	
Yes	6.933(2.363–20.343)	<0.001
Placental abruption		
No	Reference	
Yes	4.059(1.294–12.378)	0.016
PPROM		
No	Reference	
Yes	0.326(0.131–0.809)	0.016

Also, in the results of multivariate analysis of neonates born with 34- to 36^+6^-gestational-age (Table 3), we found that second labor stage, newborn weight, VBAC, fetal distress, placental abruption and PPROM were independent risk factors for the incidence of 1 min Apgar score beneath 7 (P<0.05). Among them, when the second labor stage lasted longer than 1.23 hours, the risk was increased by 4.132-fold (95% CI = 1.644–10.385; P = 0.003).

And for moderate preterm delivery, no independent meaningful factor was identified through multivariate analysis(P>0.05). So further analyses were only performed for very preterm delivery and late preterm delivery group.

### Development of predictive nomogram

With multivariable logistic analyses, we establish 4 models for very preterm delivery group. The discrimination of models was evaluated by ROC curve and its AUC value (Table 4). In model 4, we combined first and second labor stage, newborn weight and MgSO4, the AUC of it was largest (0.742, 0.670–0.805). DeLong’s test showed that there was statistically significant difference between model 2 and model 4 (P<0.0001), indicating that model4 could be a better assessment model in very preterm delivery than model 2.

**Table 4:** Models of 1-min Apgar score<7 in very and late preterm birth infants.

		AUC	95%CI	P-value
**28- to 31** ^**+6**^ **-week-old infants**				
Model1	First and second stage of labor plus Newborn weight	0.642	0.566–0.714	0.0053
Mode2	First and second stage of labor plus MgSO4	0.581	0.504–0.656	<0.0001
Mode3	Newborn weight plus MgSO4	0.642	0.566–0.714	0.0053
Mode4	First and second stage of labor plus Newborn weight plus MgSO4	0.742	0.670–0.805	Reference
**34- to 36** ^**+6**^ **-week-old infants**				
Model1	Second stage of labor plus Newborn weight plus VBAC plus Fetal Distress plus Placental abruption	0.807	0.776–0.837	Reference
Model2	Second stage of labor plus Newborn weight plus	0.754	0.719–0.786	0.0377
Model3	Second stage of labor plus Newborn weight plus VBAC	0.769	0.735–0.800	0.0866
Model4	Second stage of labor plus Newborn weight plus Placental abruption	0.765	0.731–0.796	0.0782
Model5	Second stage of labor plus Newborn weight plus Fetal Distress	0.784	0.751–0.815	0.2615
Model6	Second stage of labor plus Newborn weight plus VBAC plus Fetal Distress	0.798	0.766–0.828	0.4792
Model7	Second stage of labor plus Newborn weight plus VBAC plus Placental abruption	0.773	0.739–0.804	0.1048
Model8	Second stage of labor plus Newborn weight plus Fetal Distress plus Placental abruption	0.801	0.769–0.830	0.5672

**Fig 1:**
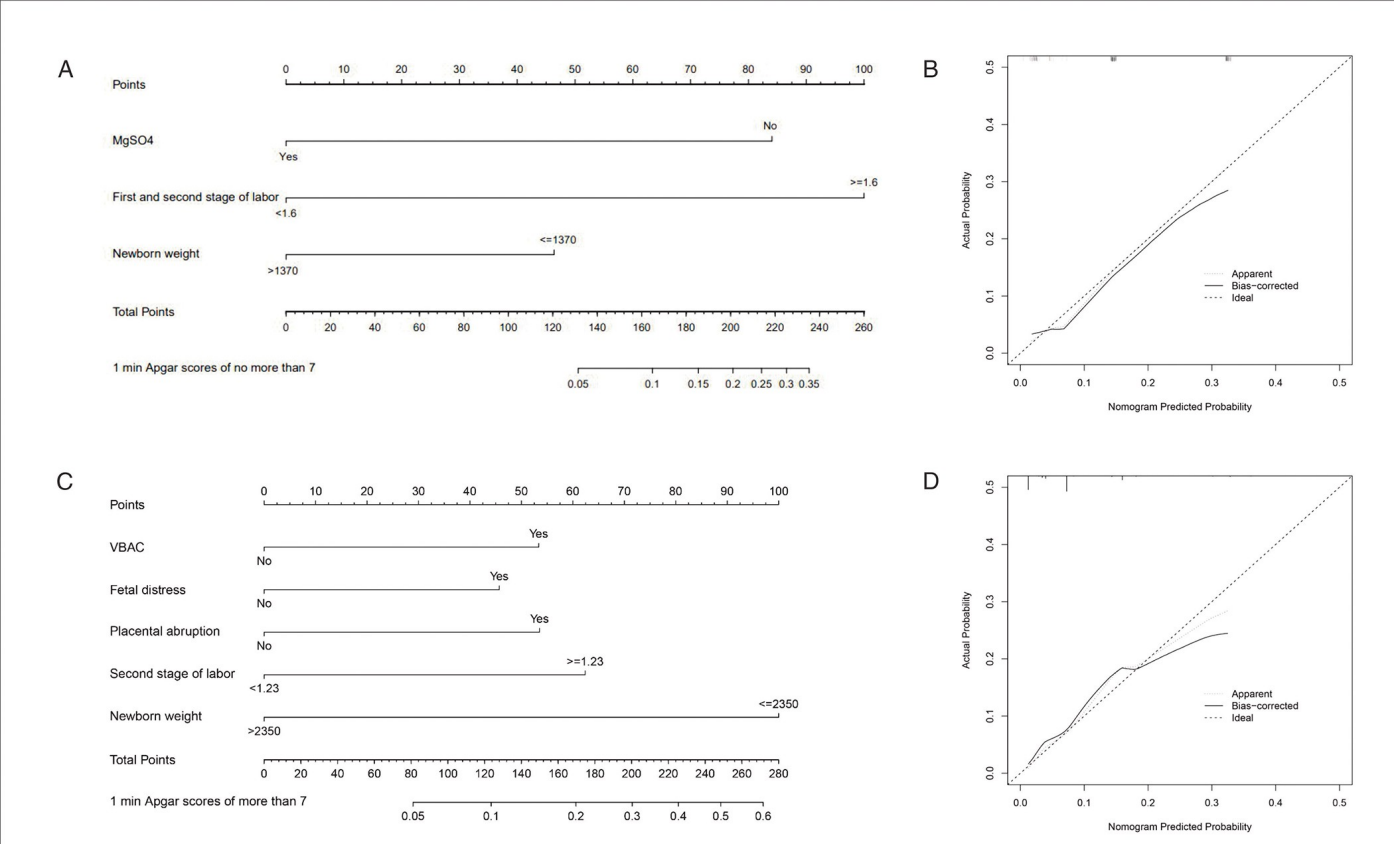
Nomogram of the predicting model. A: Nomogram of Model 4 for 1-min Apgar score less than 7 in 28- to 31+6-week-old infants; B: Calibration of Model 4 in 28- to 31+6-week-old infants; C: Nomogram of Model 1 of 1 min Apgar scores of no more than 7 in 34- to 36+6-week-old infants; D: Calibration of Model 1; MgSO4: magnesium sulfate, First and second stage of labor: the sum of the first and second labor stage(hours), Newborn weight (grams); VBAC: vaginal birth after cesarean, Second stage of labor(hours), Newborn weight(grams).

According to this, we created a nomogram prediction model for very preterm delivery with three predictors (Fig 1A). For example, for a 2000-gram (0 point) newborn whose mother didn’t administrate MgSO4 before delivery (about 84 points) and took 3 hours (about 100 points) in the first and second stages of labor, the total points are 184 points, and the likelihood of 1 min Apgar score less than 7 was about 70%-80%. The calibration curves of the prediction nomogram for the risk of 1 min Apgar score less than 7 in the patients demonstrated a good agreement (Fig 1B).

For late preterm delivery, eight models were established based on the outcomes of multivariate analyses (Table 4). And the AUC of model 1 to model 8 were as follows: 0.807, 0.754, 0.769, 0.765, 0.784, 0.798, 0.773 and 0.801 respectively. Among them, we found that the predictive value of model1 was better than model2, and the difference was statistically significant. In all forecasting models, model 1 had the highest AUC (0.807, 0.776–0.837).

Therefore, we constructed the nomogram of the prediction model with largest AUC (Fig 1C). The working instructions were the same as the above one in Fig 1A. The calibration curves of this nomogram for the risk of 1 min Apgar scores no more than 7 in 34- to 36+6-week-old infants were represented in Fig 1D.

## Discussion

The critical finding of our study was that we established the predicting model of 1 min Apgar score lower than 7 in preterm delivery. We discovered that preconception BMI, PPORM, VBAC, placental abruption, abnormal amniotic fluid, intrauterine infection, fetal distress, medicine use, labor stage and birthweight may be the potentially related factors for a low Apgar score. While taken the first and second stage of labor, newborn weight and the use of MgSO4 into consideration, the AUC of the model is 0.742, suggesting that it can predict 74.2% of 1 min Apgar score less than 7 in newborns with 28 to<32 gestational weeks. As for those born during 34 to <37 weeks, 80.7% can be identified with second labor stage, newborn weight, VBAC, placental abruption and fetal distress added to the predicting model.

A prolonged stage of labor had been reported to increase adverse neonatal outcomes like low Apgar score at 1 min or 5 min, arterial cord PH less than 7 and the chance of admission to the neonatal intensive unit in many former studies [23, 24, 29–31]. It had been demonstrated that nulliparous women with a first labor stage greater than 30 hours were more likely to have a neonate whose 5-minute Apgar score less than 7 [24]. While Wang et al found that the risk of low Apgar score increased by almost 3-fold in multiparous women whose first labor stage lasted longer than 6 hours. Additionally, as to the second stage of labor, the risk of Apgar score beneath 7 was seen to increase by 14.76 times in the subgroup longer than 3 hours in this study [29]. Another retrospective study also found that a second labor stage longer than 3 hours was correlated with a low Apgar score [32].

On the other hand, Haper found no association between the length of first labor stage and low Apgar score [30]. And the other one study figured that even the incidence of 5-min Apgar score <7 slowly increase as second labor stage prolonged, it declined greatly when it reached 4 hours [33]. Besides, there was a multiple cohort study suggesting that second labor stage may not be concerned with low Apgar score [34]. All the above study targeted at term delivery. Hence the determination of abnormal labor stage based on low Apgar score in term birth remained controversial.

While for women who delivered beyond 34 weeks, Janni W had reported that low 1-min Apgar score was associated with a second stage labor longer than 2 hours [35]. Despite that, no specific criteria of normal labor stage had been set for preterm delivery yet. While our study confirmed that a second labor stage greater than 1.23 hours in late preterm delivery may result in an over 4-fold elevated low Apgar score risk. But we found no significant difference on first stage labor between the comparison of lower Apgar score group and the normal one in very and moderate preterm delivery, whereas we could see a statistically significant difference in the combined length of first and second labor stage in this comparison(P = 0.011). Hence, we set a duration of 1.6-hour in first and second labor stage as a cutoff with a specificity of 23.8% and a sensitivity of 96.3%. With a length greater than this cutoff of labor stage, the risk of low Apgar score rose by 7.298 times.

Previous studies had described many other possible risk factors for low Apgar score at 1 min [23]. Among them, low birth weight had been regarded to concern with newborns asphyxia. Thomas Hegyi declared that birth weight can be linearly associated with Apgar score in premature newborns [36]. The universal committee for low birth weight was 2500g, while our study discovered a threshold of 1370g for very preterm delivery, and 2350g for late preterm delivery. In comparison, those who were born with a heavier birthweight may lower the risk of low Apgar score.

Another risk factor about Apgar score less than 7 in 5 minutes declared by former study was nulliparity [37], while our study witnessed no difference in primiparas and multiparas both for 1 minute Apgar score and 5 minutes Apgar score. Except for that, some precedent studies show that low Apgar score were connected to preeclampsia [38, 39]. Early diagnosis of preeclampsia (<34 weeks) and low platelet levels (HELLP) syndrome, which was defined as preeclampsia with platelet count <100,000/mm3 and serum transaminases double the averages of normal, was proved to be independent risk factor of low Apgar score at 1 minute [39]. Still, there was no difference in women with or without HDP in our study. This might be due to that we didn’t go into the further classification of hypertension disorders, which required further investigation and more detailed medical information.

### Strengths and limitations

One of the strengths of our study is that no such a visualized graph of the predictive model for 1 min Apgar score beneath 7 had been built exclusively for preterm delivery in China yet. Besides, the implication of the nomogram had been proved to be quite simple, intuitive, and practical in previous study. In addition, we still acquired a sufficient sample size with a pretty strict inclusion and exclusion committee, therefore the statistical effectiveness can be ensured. Consequently, we can achieve a more reliable result possible. And all the risk factors for the predicting models were not difficult to obtain during clinical practice, so this model should be easy to be promote in any obstetric department.

The main limitation of this study lied in the fact that the prediction value of our model had only been assessed by calibration curves and Delong’s test, external validation is still required in future research. The main predicting factor in our model is the length of labor stage. However, the definition of it is often quite subjective. It also requires professional competence and demands accumulation of clinical experience, for that those clinical parameters used in the definition, such as the effacement and dilatation of cervix can be pretty difficult to judge. Besides, due to the deficiency of moderate preterm birth cases, we fail to find independent meaningful indicators for low Apgar score in this category, further research and data collection is required in this aspect.

## Conclusion

Our study figured that a first and second stage of labor greater than 1.6 hours, birth weight smaller than 1370g and MgSO4 use history can be the optimal combination to assess the adverse outcome in very preterm neonates. While for late preterm newborns, the optimal prediction was based on the second labor stage longer than 1.23 hours, newborn weight lower than 2350 grams, VBAC, fetal distress and placental abruption. By the combination, we proposed the predicting models by the nomogram, providing the obstetrician with visible possibility of low 1-min Apgar score in preterm newborns.

## Supporting information

S1 Data (XLSX)

## Abbreviations

ROC: the receiver operating characteristic
OR: odds ratio
SPSS: statistical package for social science
SD: standard deviation
CI: confidence interval
AUC: the area under the ROC curve
BMI: prenatal body mass index
HDP: hypertension disorders
GDM: gestational diabetes mellitus
ICP: intrahepatic cholestasis of pregnancy
VBAC: vaginal birth after cesarean
PPROM: preterm premature rupture of membranes
PPH: postpartum hemorrhage
MgSO4: magnesium sulfate
NICU: the admission to the neonatal intensive care unit
